# Melanoma awareness in Jamaican patients at the University Hospital of the West Indies: A cross-sectional study

**DOI:** 10.1016/j.jdin.2023.12.006

**Published:** 2024-01-04

**Authors:** Romario D. Thomas, Althea D.C. East-Innis, Jamee J. Charles, Andrew T.W. Burton, Angela Liburd, Rodane Ruddock, Jonathan D. Ho

**Affiliations:** a Division of Dermatology, Department of Medicine, The University of the West Indies, Mona Campus, Kingston, Jamaica W.I.; b Department of Pathology, The University of the West Indies, Mona Campus, Kingston, Jamaica W.I.

*To the Editor:* Although less common in richly-pigmented populations, melanomas, particularly acral lentiginous melanomas, occur. In Jamaica, 51% are acral lentiginous melanomas.^1^ Skin of color patients are reportedly less melanoma aware.^2^^,^^3^ Late presentation contributes to poor outcomes. This cross-sectional study assesses melanoma awareness (MA) in a Jamaican/Afro-Caribbean group.

In US-based data, ∼58% of persons had some MA.^3^^,^^4^ We administered 180 questionnaires (Fig 1), 90 to dermatology and 90 to nondermatology clinic (medicine/surgery) attendees at the University of the West Indies. Questionnaires were administered to attendees agreeing to participate until sample-size achieved. Adults with no melanoma history were enrolled. Our questionnaire (partially modeled off a preexisting instrument^3^) assessed the following: (1) MA, (2) if aware, detailed knowledge, (3) information-source ranking (where one typically/historically gets information), and (4) demographic/socioeconomic data. Simply ‘hearing of melanoma’ may not influence early presentation. We hypothesize that possessing minimum specific meaningful knowledge (MK) might. Particularly, knowing its malignant nature/color/presentation/acral-predominance/detection/mortality risk potentially constitutes MK. We scored responses assigning each parameter 0/0.5/1 based on knowledge-level (Supplementary Table I, available via Mendeley at https://doi.org/10.17632/spgf32p2gk.1). MK was assigned at ≥4.5 (non-0 score per parameter).

**Fig 1:**
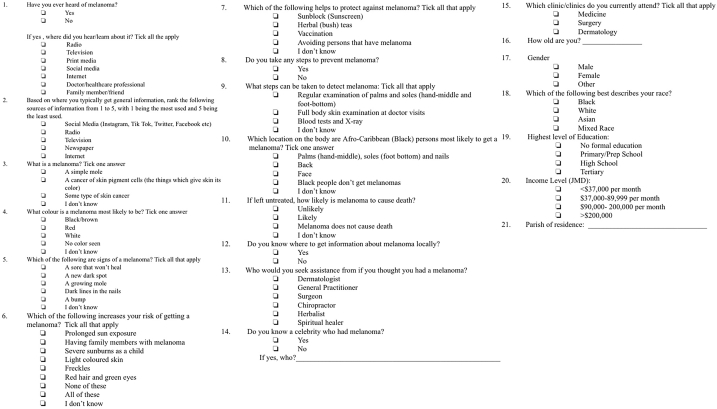
Questionnaire for evaluating melanoma awareness in a Jamaican population. *JMD*, Jamaican dollars.

Regarding socioeconomic data, education was stratified into tertiary/non-tertiary and income into relatively low/high (lower and upper 2 tiers—Fig 1). Information-sources were divided into social media/internet-based and traditional media. Descriptive statistics were generated. Association was evaluated with χ^2^/*t* tests (IBM SPSS V26).

One hundred eighty responded. Table I displays select results. Overall, 55.6% (*n* = 100) were melanoma unaware (M−) and 44.4% (*n* = 80) melanoma aware (M+); 79.5% did not know local melanoma information sources. M+ persons were significantly younger (*P* < .001). Awareness associated with tertiary education and relatively higher income (*P* < .001). No association existed between MA and sex/urbanization/dermatology clinic attendance/information-source usage.

**Table I:** Melanoma awareness in a Jamaican population: Select results

General, demographic/SE data	Overall respondents (*n* = 180)	M− group (*n* = 100)	M+ group (*n* = 80)	Association with MA (*P*-value)	Specific melanoma knowledge in M+ persons (*n* = 80) [%]
Sex (%) *n* = 180	F: 71.1	F: 69	F: 72.5	Not significant (.713)	Melanoma is a malignancy [83.8]
	M: 28.9	M: 31	M: 27.5		
	F:M ratio = 2.5:1	F:M ratio = 2.2:1	F:M ratio = 2.6:1		
Age (y) *n* = 175	Range: 18-84	Range: 21-74	Range: 18-84	**M+ respondents significantly younger than M−** **mean difference = 8.3 y** **(<.001)**	Melanoma is most commonly brown/black [62.5]
	Mean: 46.1 ± 15.06 SD	Mean: 49.7 ± 13.08 SD	Mean: 41.3 ± 16.25 SD		
Race (%) *n* = 180	Black: 88.9	Black: 92	Black: 85	Not significant (.317)	Signs of melanoma 1 sign [21.3] Multiple signs [45.0]
	Mixed: 10	Mixed: 7	Mixed: 13.8		
	White: 1.1	White: 1	White: 1.3		
POR urbanization (%) *n* = 178	High: 84.3	High: 81.8	High: 87.3	Not significant (.315)	Risk factors for melanoma 1 risk factor [22.5] Multiple risk factors [56.3]
	Low: 15.7	Low: 18.2	Low: 12.7		
Educational attainment (%) *n* = 178	No formal: 0.6	No formal: 0	No formal: 1.3	**Significantly associated. Higher proportions of tertiary educated were found in M+ group (63.8%) compared with M− group (32%)** **(<.001)**	Protective value of sunscreen [77.5]
	Primary: 8.4	Primary: 11.2	Primary: 5.0		
	Secondary: 44.4	Secondary: 56.1	Secondary: 30		
	Tertiary: 46.6	Tertiary: 32.7	Tertiary: 63.7		
Relative income bracket (%) *n* = 172	Low (tier 1-2): 61.6	Low: 73.7	Low: 46.8	**Significantly associated. M+ group had higher proportion of relatively high-income respondents (51.2%) than M− group (25%) and more relatively high-income respondents were aware of melanoma** **(<.001)**	How melanoma is detected FBSE [40] Acral + FBSE [30]
	High (tier 3-4): 38.4	High: 26.3	High: 53.2		
Clinic type (%) *n* = 180	DC: 50	DC: 45	DC: 56.3	Not significant (.134)	Acral location in Afro-Caribbean persons [33.8]
	NDC: 50	NDC: 55	NDC: 43.8		
Primary sources of general information (%) *n* = 179	SMIB: 67.6	SMIB: 61 TM: 37	SMIB: 76.3 TM: 23.7	Not significant (.07)	Aware of mortality risk of untreated melanoma [56.3]
	TM: 31.3				
	Equal SMIB/TM: 1.1				

Bolded text in column 5 represents statistically significant associations. The column 6 additionally reports detailed melanoma knowledge in melanoma aware persons.

*DC*, Dermatology clinic; *F*, female; *FBSE*, full body skin examination; *M*, male; *M+*, melanoma-aware group; *M−*, melanoma-unaware group; *MA*, melanoma awareness; *NDC*, nondermatology clinic; *POR*, parish of residence; *SE*, socioeconomic; *SMIB*, social media/internet-based resources; *TM*, traditional media.

Although most M+ respondents (83.5%) identified melanoma as skin cancer, 37.5% did not know/incorrectly selected its likely color, 65% were ignorant of acral predominance in Afro-Caribbean persons and only 56.3% thought untreated melanoma likely to kill (Table I); 35% achieved MK (score ≧4.5). Analyzing MK as the end point, association with income/education was lost (*P* = .67/0.054). MK-achievers were younger (34.2 ± 16.23 SD years) than their counterparts (45.1 ± 15.09 SD years, [*P* = .003]); 50% reported social media/internet-based, 45% doctors, 30% family/friends, and 23.8% traditional media as contributing to MA. Given his potential celebrity leverage, we evaluated M+ awareness of Bob Marley’s melanoma diagnosis. Only 25% (*n* = 20) endorsed knowing a celebrity with melanoma; 80% identifying Marley.

Most of our sample was melanoma unaware. Our MA (44.4%) is lower than recent data (United States) where 57.6% of richly-pigmented participants identified melanoma as skin cancer.^3^ Younger age, tertiary education, and relatively high-income significantly associated with MA.

Population-relevant acral lentiginous melanoma–focused information was less known than UV-related melanoma information (Table I) suggesting knowledge acquisition from international, not local educators. Although hypothetical, we advance the logic that MK potentially increases the chance of early diagnosis. Only 35% of M+ persons achieved MK. The data suggest a relative lack of correct/detailed knowledge in aware persons.

Targeted education may increase MA. Utilization of typical information gathering sources, celebrity leveraging (perhaps Marley), and capitalizing on dermatology visits for population-relevant education are suggested. Limitations include urban-predominant respondents, a single-center population and use of unvalidated instruments.

## Conflicts of interest

None disclosed.
